# *Datura stramonium *L. poisoning in a geophagous child: a case report

**DOI:** 10.1186/1865-1380-4-31

**Published:** 2011-06-15

**Authors:** Asma Bouziri, Asma Hamdi, Aida Borgi, Sarra Bel Hadj, Zohra Fitouri, Khaled Menif, Nejla Ben Jaballah

**Affiliations:** 1 Paediatric intensive care unit, Children's Hospital of Tunis, Tunisia

## Abstract

*Datura stramonium *L. (DS) is a wild-growing plant widely distributed and easily accessible. It contains a variety of toxic anticholinergic alkaloids such as atropine, hyoscamine, and scopolamine. Voluntary or accidental ingestion can produce severe anticholinergic poisoning. We report an unusual case of DS intoxication occurring in a geophagous young child after accidental ingestion of the plant. Our case is original because of the young age of the victim and the underlying geophagia facilitating the occurrence of poisoning.

## Introduction

*Datura stramonium *L (DS) is a hallucinogenic plant widely found in urban and rural areas. Toxicity from this plant, containing tropane alkaloids, manifests as a classic anti-cholinergic poisoning [1,2]. Most cases of DS poisoning reported in the literature occurred among teenagers after voluntary ingestion of the plant for its hallucinogenic and euphoric effects [1,3-6]. This report illustrates an unusual case of DS poisoning occurring in a geophagous 3.5-year-old child after accidental ingestion of the foliage of the plant with earth.

## Case report

A 3.5-year-old girl was brought to the emergency department by her parents for excitation, delirium, and hallucinations occurring within the hour following accidental ingestion of DS. She had a medical history of geophagia complicated by iron deficiency anemia, which had been treated 6 months ago. The child had the habit of eating earth, and she had ingested the foliage of the toxic plant with earth, in the presence of her mother, during a walk in the fields. On the initial physical examination, her vital signs were: temperature 37.7°C, pulse rate 190 beats/min, respiratory rate 25 breaths/min, blood pressure 104/72 mmHg, and oxygen saturation 99% on room air. Her mouth was dry. Facial and truncal skin was normal, and flushing was not detected. She had a Glasgow Coma Scale score of 11/15. She was agitated and aggressive with purposeless movements, delirium, and hallucinations: she saw wild animals, a man who wanted to beat her, and various other things. Her pupils were widely dilated and not reactive to light. The neurological examination also noted hypertony with exaggerated deep tendon reflexes, clonus of the feet, and tremulousness. Meningeal irritation signs were not detected. Abdominal distension and urinary retention were noted. She was transferred to the pediatric intensive care unit. Gastric decontamination with nasogastric lavage and activated charcoal via a gastric tube was rapidly performed after admission. Intravenous (IV) fluids and diazepam (0.5 mg/kg) were administered. The patient remained agitated and was given two more doses of 0.5 mg/kg IV diazepam over the first 4 h of hospitalization. The biological results showed normal serum levels of urea, creatinine, glucose, Na, and K, and calcemia. White blood cell (WBC) and platelet levels were normal, but she presented hypochromic microcytic anemia with a hemoglobin level of 7.8 g/dl. Aspartate aminotransferase, alanine aminotransferase, creatine kinase, lactic dehydrogenase, and gamma glutamyl transpeptidase levels were normal. Prothrombin time and INR were 76% and 1.1, respectively. The clinical course was favorable. Repeated neurological examination revealed a gradual improvement of her state of consciousness with disappearance of tachycardia, mydriasis, delirium, and agitation. On the second day of hospitalization, her neurological examination was normal, and she was discharged home under oral iron treatment.

## Discussion

*Datura stramonium *L. (DS) is a wild-growing herb known as Jimson weed [1]. It also has several slang names; the most common in our context is "sak el ghoul." The whole plant, particularly the foliage and seeds, is toxic because it contains the tropane alkaloids atropine, L-hyoscyamine and L-scopolamine, which are responsible for anticholinergic syndrome resulting from the inhibition of central and peripheral muscarinic neurotransmission by these toxic components [1]. Causes of DS intoxication include medication overdose, improper preparation of edible vegetables, deliberate abuse as a hallucinogen, use for homicide or robbery (we approve this correction), and accidental intoxication [2]. Most of the cases reported in the literature occurred among teenagers after voluntary ingestion of the plant for its hallucinogenic and euphoric effects [1,3-6]. To our knowledge, our patient is the youngest case of DS poisoning reported in the literature. The occurrence of this poisoning in our patient was facilitated by an abnormal dietary behavior called geophagia or pica, characterized by a perverted appetite for substances inappropriate for consumption, such as clay and earth [7]. Pica can lead to serious health complications such as iron deficiency anemia, electrolyte and metabolic disorders, parasitic infections, tooth wear, and intestinal obstruction. This condition has been observed in men and women of all ages and ethnicity, but is more prevalent among lower socioeconomic classes, pregnant women, and small children [7]. Our geophagous child developed a typical form of DS poisoning characterized mainly by a toxic delirium occurring rapidly after ingestion. Typical symptoms of DS intoxication are those of atropine intoxication, which are dry skin and mucosa, flushing, mydriasis, sinus tachycardia, hyperpyrexia, decreased bowel sounds, urinary retention, and neurological disorders with ataxia, impaired short-term memory, disorientation, confusion, hallucinations (visual and auditory), psychosis, agitated delirium, seizures, and coma. In severe forms, respiratory failure and cardiovascular collapse have been reported [1-6]. Rarely, rhabdomyolysis and fulminant hepatitis have also been described [8]. DS toxicity usually occurs within 60 min after ingestion, and clinical symptoms may persist for 24 to 48 h because the anticholinergic effects delay gastric emptying, resulting in a prolonged duration of action. Children have a special susceptibility to atropine toxicity; even a small amount may produce severe central nervous system manifestations [9]. Despite the young age of our patient, a rapid improvement of the neurological manifestations was obtained, probably because the diagnosis was evident and gastric decontamination was carried out soon after ingestion. The diagnosis of DS poisoning is essentially clinical, but tropane alkaloids may be detected by gas chromatography and mass spectrometry [1]. The treatment is essentially supportive and consists of gastric decontamination with activated charcoal by mouth or tube, control of agitation with benzodiazepines, and hyperpyrexia control (fluids and other cooling measures). Tachycardia usually responds to crystalloids [10]. Although physostigmine is the antidote for anticholinergic toxicity, its use is controversial despite recent reports of it being a safe treatment. Physostigmine is recommended when the patient has severe agitation or psychosis not controlled with bezodiazepines or has intractable seizures or tachydysrhythmias with hemodynamic compromise [1]. Phenothiazines for agitated delirium should be avoided due to their anticholinergic properties, and barbiturates can be administered in the case of seizures refractory to benzodiazepines [1,10]. The prognosis of DS intoxication is usually favorable, as in our case, but it may be fatal, especially during massive intoxications meant to be autolytic or the result of toxicomania [6].

## Conclusion

We report an unusual case of acute DS intoxication in a 3-year-old girl who presented with toxic delirium 1 h after ingesting the foliage of the DS plant. Our case is original because of the young age of the victim and the underlying geophagia facilitating the occurrence of poisoning.

## Consent

Written informed consent was obtained from the patient's parents for publication of this case report. A copy of the written consent is available for review by the Editor-in-Chief of this journal.

## Competing interests

The authors declare that they have no competing interests.

## Authors' contributions

AB, AH and AB drafted the manuscript, SBH and ZF participated in the collection of the bibliography, KM and NBJ participated in the design of the study and coordination of the manuscript. All authors read and approved the final manuscript.
